# Exoskeleton rehabilitation robot training for balance and lower limb function in sub-acute stroke patients: a pilot, randomized controlled trial

**DOI:** 10.1186/s12984-024-01391-0

**Published:** 2024-06-08

**Authors:** Yuting Zhang, Weiwei Zhao, Chunli Wan, Xixi Wu, Junhao Huang, Xue Wang, Guilan Huang, Wenjuan Ding, Yating Chen, Jinyu Yang, Bin Su, Yi Xu, Zhengguo Zhou, Xuting Zhang, Fengdong Miao, Jianan Li, Yongqiang LI

**Affiliations:** 1 The First Affiliated Hospital of Nanjing Medical University, Nanjing, China; 2 Wuxi Central Rehabilitation Hospital, The Affiliated Mental Health Center of Jiangnan University, Wuxi, Jiangsu China; 3 Nanjing Medical University, Nanjing, China; 4 Nanjing Sport Institute, Nanjing, China; 5 Wuxi MaxRex Robotic Exoskeleton Limited, Wuxi, China

**Keywords:** Rehabilitation robot, Stroke, Balance function, Lower limb function

## Abstract

**Purpose:**

This pilot study aimed to investigate the effects of REX exoskeleton rehabilitation robot training on the balance and lower limb function in patients with sub-acute stroke.

**Methods:**

This was a pilot, single-blind, randomized controlled trial. Twenty-four patients with sub-acute stroke (with the course of disease ranging from 3 weeks to 3 months) were randomized into two groups, including a robot group and a control group. Patients in control group received upright bed rehabilitation (*n* = 12) and those in robot group received exoskeleton rehabilitation robot training (*n* = 12). The frequency of training in both groups was once a day (60 min each) for 5 days a week for a total of 4 weeks. Besides, the two groups were evaluated before, 2 weeks after and 4 weeks after the intervention, respectively. The primary assessment index was the Berg Balance Scale (BBS), whereas the secondary assessment indexes included the Fugl-Meyer Lower Extremity Motor Function Scale (FMA-LE), the Posture Assessment Scale for Stroke Patients (PASS), the Activities of Daily Living Scale (Modified Barthel Index, MBI), the Tecnobody Balance Tester, and lower extremity muscle surface electromyography (sEMG).

**Results:**

The robot group showed significant improvements (*P* < 0.05) in the primary efficacy index BBS, as well as the secondary efficacy indexes PASS, FMA-LE, MBI, Tecnobody Balance Tester, and sEMG of the lower limb muscles. Besides, there were a significant differences in BBS, PASS, static eye-opening area or dynamic stability limit evaluation indexes between the robotic and control groups (*P* < 0.05).

**Conclusions:**

This is the first study to investigate the effectiveness of the REX exoskeleton rehabilitation robot in the rehabilitation of patients with stroke. According to our results, the REX exoskeleton rehabilitation robot demonstrated superior potential efficacy in promoting the early recovery of balance and motor functions in patients with sub-acute stroke. Future large-scale randomized controlled studies and follow-up assessments are needed to validate the current findings.

**Clinical trials registration:**

URL: https://www.chictr.org.cn/index.html.Unique identifier: ChiCTR2300068398.

**Supplementary Information:**

The online version contains supplementary material available at 10.1186/s12984-024-01391-0.

## Introduction

Stroke is the second leading cause of mortality and the third leading cause of disability worldwide [1]. During the recent decades, owing to rapid advancement in stroke treatment, global stroke mortality showed a significant decline [2]. Therefore, the total population of stroke survivors has increased and large population of stroke survivors would live with persistent dysfunctions. According to relevant statistics, more than 70% of stroke survivors will be left with motor, sensory, cognitive, and speech dysfunctions to varying degrees, which have resulted in the loss of personal labor force and posed a heavy burden on both the families and the society [3].

Balance, defined as the ability to maintain stable posture across diverse environments and conditions, is fundamental to all human static and dynamic activities [4]. Balance dysfunction may occur in more than 80% of stroke survivors, and is characterized by poor trunk control, insufficient muscle strength in the lower limbs, poor weight bearing in the affected lower limbs and slower walking speed [5, 6]. Such dysfunction can adversely affect mobility and quality of life [7]. Compromised balance is associated with an increased risk of falls [8], which may lead to restricted activities, physiological deconditioning, diminished independence, heightened fear of falling, and a higher incidence of subsequent falls [9]. In addition, balance is considered as an important factor for the walking ability of patients and is an important predictor of whether a patient will be able to walk independently [10]. Therefore, improving balance function and balance response strategies are the important goals in stroke rehabilitation programs [11].

Robotic training, characterized by high repetition, dosage, and intensity, has emerged as a cost-effective intervention in recent years [12]. Currently, exoskeleton rehabilitation robots ahave gained remarkable attention in recent years lower limb rehabilitation in stroke survivors [13]. While definitive evidence remains elusive regarding the superiority of exoskeleton-assisted training over conventional therapy, various studies have suggested it may enhance gait, ambulatory capabilities, balance, reduce muscle spasticity in the lower limbs, and improve cardiorespiratory fitness in individuals post-stroke [14, 15]. A meta-analysis has indicated that exoskeleton-assisted gait training is either beneficial or comparable to traditional rehabilitation methods for recovering gait and balance in stroke patients [16].

In this study, we utilized the REX robotic exoskeleton (REX Bionics PLC, London, UK), a self-stabilizing device that allows for the performance of upper body exercises in an upright position without the need for additional upper body support or balance aids, such as crutches or walking frames. This represents a significant deviation from other rehabilitation robot paradigms [17, 18]. Currently, there is only one study demonstrating the good feasibility, safety, and acceptability of the REX rehabilitation robot for the physical activity and upper body movement training in patients with spinal cord injury [19]. Therefore, the objective of this study is to investigate the effectiveness of REX exoskeleton rehabilitation robot training on the balance and lower limb function in patients with stroke in the sub-acute rehabilitation phase. Notably, we focused on determining whether REX exoskeleton rehabilitation robot training was superior to dose-matched conventional training with regard to the balance and lower limb function in patients with sub-acute stroke.

## Methods

### Trial design

This was a pilot, single-blind, randomized controlled clinical trial, in which the assessor was blinded based on the CONSORT statement. The trial protocol was approved by the Ethics Committee of the Wuxi Mental Health Center (No. WXMHCIRB2022LLky038) and was registered in the Chinese Clinical Trial Registry prior to the study (unique identifier: ChiCTR2300068398). All subjects signed a written informed consent form before initiating the trial.

### Setting, recruitment and participants

Patients with sub-acute stroke who received rehabilitation treatment at the Department of Rehabilitation from Tongren Rehabilitation Hospital in Wuxi between June and December 2022 were carefully chosen in this study. The patient inclusion criteria were as follows: (1) patients with post-stroke hemiplegia conforming to the ‘Key Points for Diagnosis of Various Cerebrovascular Diseases’ revised by the 2019 National Cerebrovascular Disease Academic Conference of the Chinese Medical Association [20], which was diagnosed based on head CT or MRI; (2) patients with first-onset stroke (with the course of disease ranging from 2 weeks to 3 months); (3) patients aged 40–75 years; (4) patients with Brunnstrom of the lower limb of the hemiplegic side ≤ 3 stage; (5) patients with lower limb muscle spasm ≤ 2 grade; (6) patients with Berg Balance Scale (BBS) score ≤ 20 points; (7) patients with stable vital signs and non-progressive neurological symptoms; and (8) patients who were able to understand and communicate in a simple manner (Minimum Mental State Examination, MMSE score ≥ 21 points). Exclusion criteria included: (1) patients with severe arthritis or other serious musculoskeletal diseases; (2) patients with systemic immune diseases, blood system diseases or other diseases; (3) patients with severe mental abnormalities, who were unable to cooperate with the completion of treatment or those with poor compliance; (4) patients with orthostatic hypotension, deep vein thrombosis of the lower extremities, unstable hypertension and severe heart or cardiopulmonary diseases who could not participate in sports; and (5) patients who refused to sign the informed consent.

### Procedures

The sample size calculation was conducted using G*Power 3.1.7 (http://www.gpower.hhu.de/). The effect size was estimated using our pilot data regarding in BBS after training (experimental group vs. control group: 36.84 ± 12.11 vs. 22.58 ± 9.99) would be able to reveal a large effect size of Cohen’s d = 1.28, at a power of 0.8 and an αlevel of 0.05 assuming a non-directional hypothesis. Thus, in the current study, a large effect size f = 0.54 was assumed in the T test model, with an α value of 0.05, power of 0.8, and an attrition rate of 10%, the minimum required sample size was estimated to be 24 subjects for this study.

Subsequently, these patients were randomly allocated to either a robot group or a control group at a ratio of 1:1 using a computer-generated random number table. Randomized grouping was implemented by a statistician who was not involved in this study, and group concealment was retained until the allocation was completed. All assessors were blinded to the group assignments throughout the study.

### Interventions

Patients in both groups participated in regular rehabilitation training (40 min/session, 2 sessions/day, 5 days/week for a total of four weeks) tailored to individual functional capabilities. This training included transfers, sit-to-stand, static and dynamic balance training, walking training, and aerobic training.

**Fig. 1:**
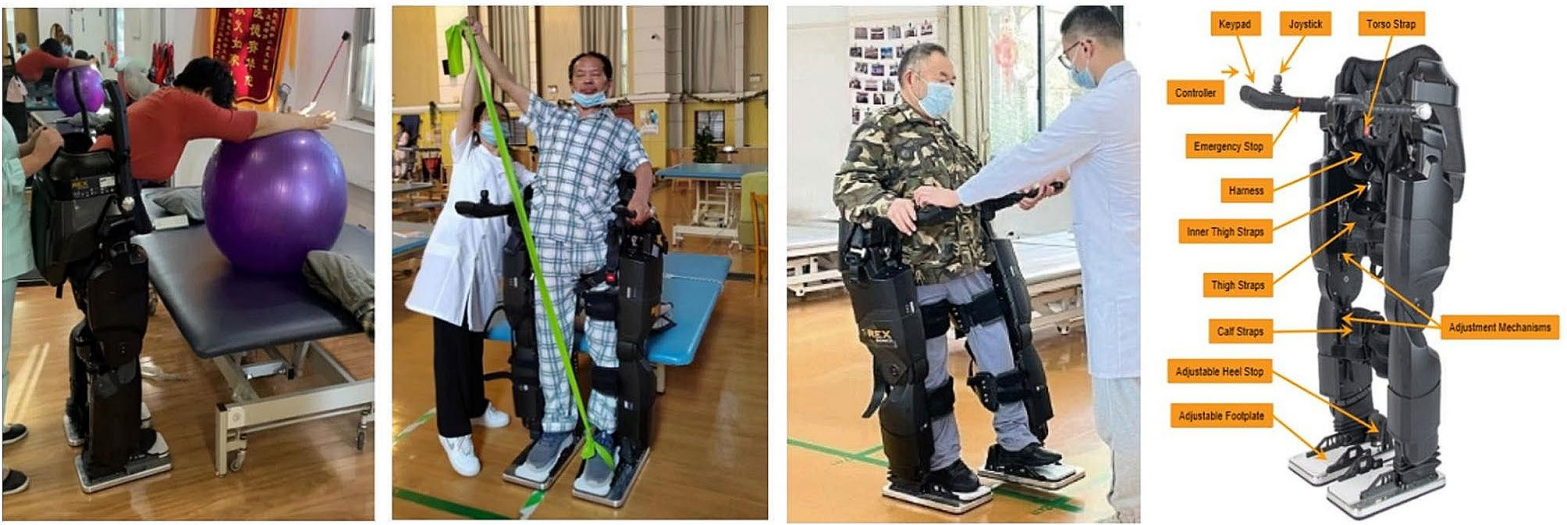
(**A**): Standing balance training. (**B**): Elastic band resistance training. (**C**): Lower limb function training. (**D**): Rex robot structure

Patients in the robot group further received REX rehabilitation exoskeleton rehabilitation robot training on the basis of conventional rehabilitation treatment. This supplementary program comprised: (1) Standing position activity training, including: (a) A Bobath ball was placed on the treatment bed and the patient was worn in the REX exoskeleton rehabilitation robot, which used both upper limbs to push the ball and guide the trunk in an anterior-posterior direction on the frontal plane and in a left-right direction on the horizontal plane, as shown in Fig. 1A. (b) The patient performances in reaching for objects (occupational therapy) at different heights (table and cabinet) and in multiple directions (in front of the body, to the left, and to the right of the body) in the REX exoskeleton rehabilitation robot; (2) Elastic band resistance training: in which the patient war worn in the REX exoskeleton rehabilitation robot, the Thera-Band elastic band was bound onto the patient’s lower limb foot, and then the patient was instructed to perform upper limb resistance training on the healthy side, so as to promote the lower limb and trunk extension on the affected side. The way of training referred to the upper limb PNF diagonal spiral pattern, as displayed in Fig. 1B; (3) Lower limb function training: the patient was worn in the REX exoskeleton rehabilitation robot and used the affected lower limb for single leg weight bearing, lateral stride, unilateral repeated stride, squatting training as well as alternating stride training of the lower limbs on the right and left sides in the anterior and posterior direction, as shown in Fig. 1C. There were three training programs in total, each of which was performed in 20 min for 60 min/day and 5 days per week for 4 weeks.

In the control group, upright bed standing training was added on the basis of conventional rehabilitation treatment, including (1) The patient was in the standing position on the upright bed, and the upper limbs were trained to different heights and directions for working activities; (2) The patient was in a standing position on the upright bed, the healthy lower limb was lifted, and the affected lower limb was trained to bear weight on one leg. The training frequency was 60 min/day for 5 days per week for 4 weeks.

### REX exoskeleton rehabilitation robot

The REX exoskeleton rehabilitation robot (Rex, New Zealand, Fig. 1D) was a wearable, self-stabilizing dynamic exoskeleton robot. Movement control was simple and easily operated through its control lever and control panel. Its bionic leg parts were controlled by the network composed of 29 micro-controllers, which perfectly coordinated with the lower limb movement. When the patient stood or moved, there was no need to provide additional supporting auxiliary tools (such as crutches) to maintain the balance. Once fitted with the REX exoskeleton, patients can mobilize their arms without restriction. The robot auxiliary rehabilitation therapy (robot-assisted physiotherapy, RAP) program, developed by REX and therapists, facilitated functional movement exercises, including weight-bearing, steps, sitting, squatting, bow steps, as well as upper limb and trunk activities aimed at enhancing aerobic capacity.

### Assessments

Assessments were conducted before the intervention (T0), 2 weeks after the intervention (T2), and 4 weeks after the intervention (T4). The assessors included therapists who were independent of the study and did not participate in the treatment of the study. These assessors had more than 5 years of clinical experience, were proficient in and applied the assessment-related tools and methods. The assessors were blinded to the group assignments.

### Primary outcome

The primary outcome was the Berg Balance Scale (BBS), which observed the change in the indicator after 4 weeks of intervention compared with that in the pre-intervention period. BBS is comprised of 14 items, each of which adopts a 5-point scoring system from 0 to 4 points, with a total score of 56 points. A higher score indicates the better balance ability. The test achieves excellent intra-examiner reliability and validity in the assessment of stroke balance function [21]. The minimal clinical difference in BBS in the sub-acute phase is 5 points [22].

### Secondary outcomes

The secondary outcomes included the Fugl–Meyer lower-extremity motor function scale (FMA-LE), the postural assessment scale for stroke (PASS), the activities-of-daily-living assessment scale (Modified Barthel Index, MBI), Tecnobody balance testing and lower-extremity surface electromyography (sEMG).

FMA-LE is a comprehensive score for the reflex activity, joint activity, coordination ability and speed of the lower limbs. The total score ranges from 0 to 34 points, and the higher score indicates the better motor function of the lower limbs [23]. PASS can be adopted to evaluate the recumbent position change ability, lying–sitting transfer ability, sitting–standing position transfer ability, sitting balance, standing position balance, as well as bending and picking up objects of patients with stroke. The minimum score for each item is 0 point whereas the maximum score is 3 points (0–36 points), and the higher scores indicate better postural control and balance abilities [24]. MBI can be applied in evaluating the daily life ability of patients, including eating, bathing, grooming, dressing, toilet control, using the toilet, transfer, walking on level ground and going up and down the stairs. The total score is 100 points, and a higher score stands for the better daily life ability. Tecnobody balance tester is mainly used to evaluate the static stability and stability limits in the upright position. The lower static stability evaluation score represents the smaller body swing and the better motor control and balance function of the patients. Moreover, stability limit was evaluated by the acquisition rate of the target object in this study. During the test, patients were required to try their best to shift the center of gravity to the eight quadrants to reach the corresponding target object and keep it for a certain period. The greater stability limit stands for the better surface balance function [25].Surface EMG signals were collected from the lower limb muscles, including the rectus femoris, biceps femoris, tibialis anterior, and gastrocnemius, using a FreeEMG 300 wireless surface EMG system (Bettisco Technology, Italy). Before data acquisition, we provided detailed instructions to the patients to ensure they understood the commands and movements required. The skin over the target muscles was exposed, shaved if necessary, and cleaned with 75% alcohol to remove grease and keratin, reducing electrical resistance. After the skin dried, sensors and electrodes were attached to the designated positions.During the test, patients were instructed to perform a maximum voluntary contraction for 10 s after hearing the command “start.” Each muscle was tested three times with a 5-minute rest interval between trials to prevent muscle fatigue from influencing the results. Data were stored on a computer via the receiver. The raw EMG signals were processed using MegaWin V3.0 software, which included full-wave rectification, smoothing, and window width adjustments. The “region of interest” was selected, and the software was used to calculate the average RMS EMG and iEMG values for the specified region. RMS is calculated by the integral myoelectricity divided by the time of monitoring the integral myoelectricity. It compares the general level of muscle discharge within a certain period. Some scholars have suggested that it is related to the number of motor units recruited and the synchronization of muscle fibre discharge [26]. iEMG refers to the total amount of motor unit discharge generated by muscle activity during a specific period, in other words, the magnitude of iEMG can reflect the number of motor units participating in muscle activity and the discharge value of each motor unit at the same time [27]. The reliability and validity of the above assessment tools and methods in patients with stroke have been confirmed [28]. In addition, the vital signs (heart rate, blood pressure, and oxygen saturation), as well as subjective discomfort of the patients were recorded during training.

### Statistical analysis

SPSS software version 22.0 (IBM Corp., Armonk, NY, USA) was used for statistical analysis. Shapiro-Wilk was adopted to test the normality of all parameters. T-test was utilized for measurement data, chi-square test for count data, and rank sum test for rank data. The primary and secondary assessment indicators at pre-intervention (T0) and 4 weeks after intervention (T4) were compared by paired t-tests within groups and by independent t-tests between groups. Meanwhile, comparisons within and between groups at the three time points (T0, T2, T4) were analyzed based on the repeated measures ANOVA. For significant interactions, post hoc analyses and multiple comparisons were performed by Bonferroni adjustment to adjust for the probability of type I error. A Greenhouse-Geisser correction was performed when Mauchly’s test of sphericity revealed a clear violation of this assumption. A p-value of less than 0.05 was considered indicative of statistical significance.

### Result

#### Flow of participant selection

**Fig. 2:**
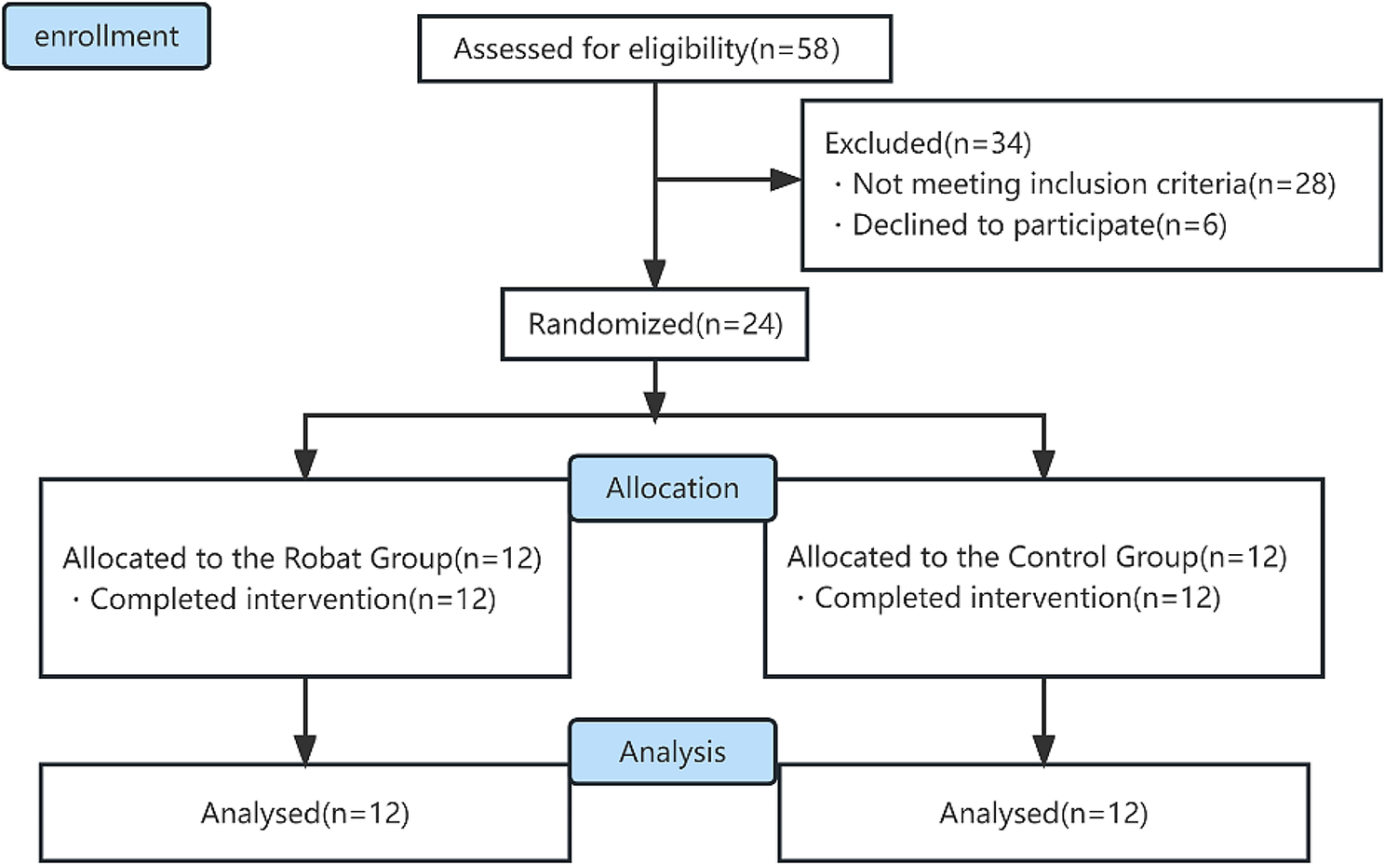
Altogether 58 patients were screened for eligibility and finally 24 of them were enrolled for analysis

From June to December 2022, a total of 58 patients were screened for participation in this study. Of these, 28 did not meet the inclusion criteria, and another 6 withdrew for personal reasons. Finally, altogether 24 patients met our eligibility criteria and were randomly assigned to the robot (*n* = 12) and control groups (*n* = 12). All enrolled patients completed the study and their data were included in the final analysis (Fig. 2). No adverse events were reported in this study and patients did not report any discomfort.

### Baseline data

The baseline characteristics of all participants are presented in Table 1. The mean age was 63.67 (SD 8.44) years, and the mean duration of stroke was 49.58 (SD 26.45) days. Among the participants, 11 had a stroke on the left side and 13 on the right. No significant differences were found between the two group in terms of gender, age, duration, type or side of stroke (*P* > 0.05).

**Table 1:** Demographic and clinical characteristics of patients at baseline (*N* = 24)

Variable		Robot group (*n* = 12)	Control group (*n* = 12)	*P* value
Age (y)		63.50 ± 8.97	63.83 ± 8.28	0.92
Gender, n (%)	Male	10 (83.33%)	8 (66.67%)	0.35
	Female	2 (16.67%)	4 (33.33%)	
Stroke onset duration, (d)		46.67 ± 24.62	52.50 ± 28.96	0.53
Stroke type, n (%)	Ischemic	5 (41.67%)	7 (58.34%)	0.41
	Hemorrhagic.	7 (58.34%)	5 (41.67%)	
Side of stroke, n (%)	Left	6 (50%)	5 (41.67%)	0.68
	Right	6 (50%)	7 (58.34%)	

Values are presented as mean (SD). *Statistically significant (*P* < 0.05).

### Outcome measures

As observed from Table 2, our results showed a significant improvement in the functional assessment of BBS scores following 4 weeks of intervention in both groups. The robot group’s BBS scores improved from a mean of 10.25 ± 6.47 pre-intervention to 32.5 ± 13.42 post-intervention, while the control group’s scores increased from a mean of 10.92 ± 4.98 to 20.58 ± 12.05 post-intervention. Additionally, the improvement was significantly greater in robot group than in control group (*p* = 0.032).

Moreover, postural control and balance function of the patients were comprehensively assessed using the Stroke Postural Scale PASS, which demonstrated significant improvements in both the robot and control groups after 4 weeks of intervention (pre-intervention: 16.33 ± 6.51 for robot group and 15.17 ± 5.34 for control group; after 4 weeks of intervention: 30.08 ± 7.74 for robot group and 21 ± 6.59 for control group). When compared between two groups, a greater improvement was observed in robot group than in control group (*p* = 0.005).

FMA-LE reflects the lower limb motor function in stroke patients. Post-intervention, both groups showed significant enhancements in FMA-LE scores; the robot group improved from 12.33 ± 4.85 to 19.42 ± 6.73 and the control group from 11.42 ± 4.06 to 16.58 ± 6.6. However, there was no significant difference between pre-intervention and after 4 weeks of intervention in these two groups (*p* > 0.05).

In addition, MBI, an indicator assessing the independence in daily living activities, showed a significant difference in both robot and control groups between after 4 weeks of intervention and pre-intervention. Nevertheless, the improvements did not differ significantly between the robot and control groups (*P* > 0.05).

Furthermore, the Tecnobody Balance Tester was used to assess the patient’s static balance stability under the eyes open and closed conditions and the stability limit for dynamic assessment. After 4 weeks of intervention, the robot group demonstrated significant improvements in static balance, as evidenced by reductions in the length of the movement trajectory with eyes open (from 229.25 ± 83.71 to 158.25 ± 69.18) and with eyes closed (from 223.25 ± 73.38 to 160.25 ± 63.64). Additionally, the area of movement decreased with eyes open (from 223.58 ± 282.42 to 47.58 ± 46.6) and with eyes closed (from 145.5 ± 138.59 to 36.42 ± 38.1). Meanwhile, In the control group, there were significant improvements in the length of movement trajectory with eyes open (from 239.08 ± 85.26 to 174.5 ± 43.72) and the area of movement with both eyes open (from 237.78 ± 167.87 to 186.64 ± 154.93) and eyes closed (from 152.19 ± 132.01 to 104.98 ± 116.25). The static balance stability improved in both groups after the 4-week intervention. A significant difference between the groups was observed in the area of movement with eyes open, with the robot group showing greater improvement than the control group (*p* = 0.011). For dynamic balance, both groups showed significant improvement in stability limit measures after the intervention, with the robot group improving from 39.29 ± 14.97 to 69.89 ± 11.85 and the control group from 42.7 ± 9.33 to 59.6 ± 9.65. The improvement was greater in the robot group compared to the control group (*p* = 0.03).

As demonstrated by the results of lower limb sEMG analysis, there were significant improvements in RMS and iEMG of rectus femoris and biceps femoris in the robot group and control group, but not in RMS and iEMG of tibialis anterior and gastrocnemius muscles. In addition, there was no significant difference in rectus femoris, biceps femoris, tibialis anterior or gastrocnemius muscles in robot group compared with control group (*p* > 0.05).

**Fig. 3:**
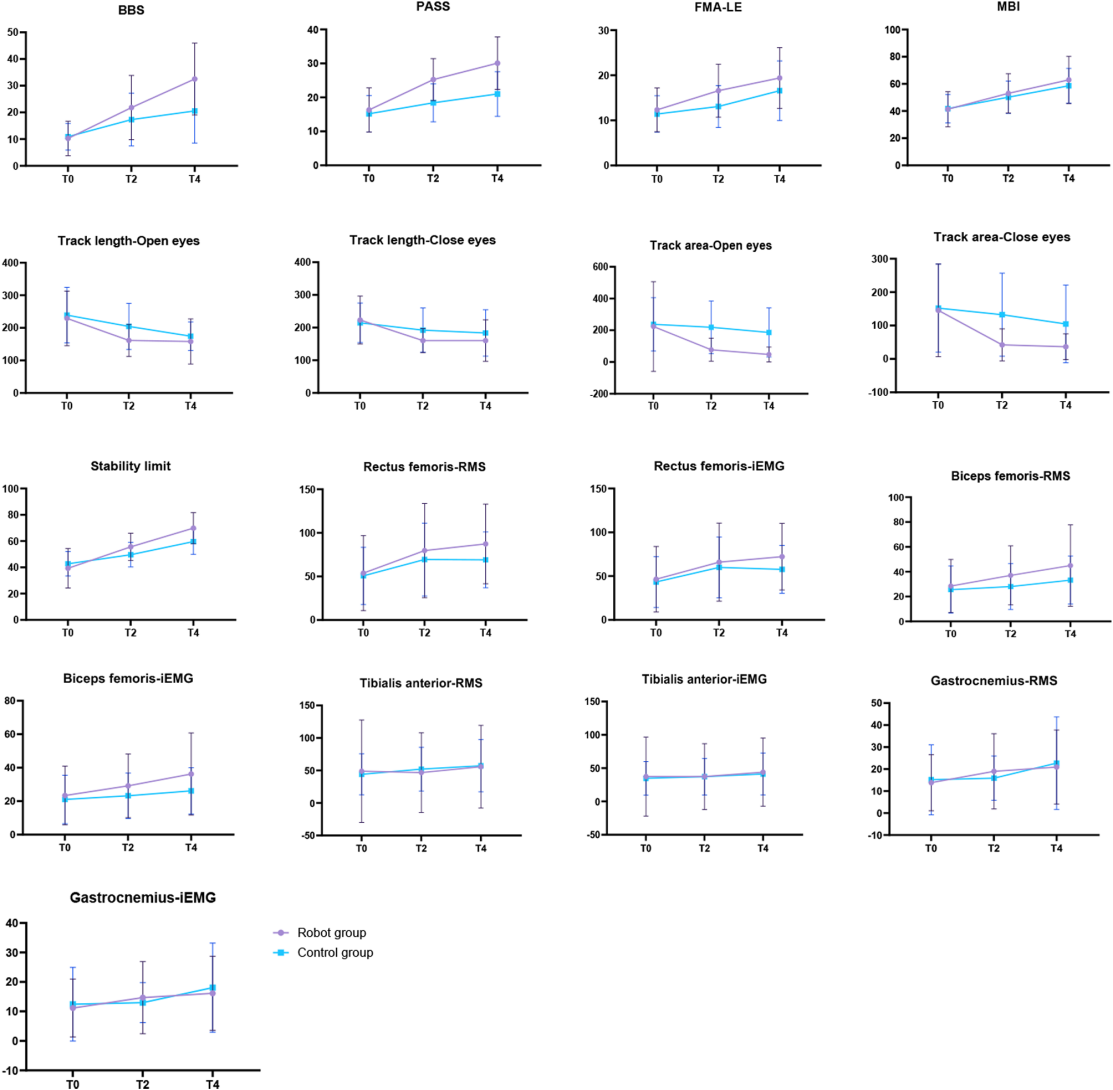
Changes in assessment metrics before intervention (T0), after 2 weeks of intervention (T2), and after 4 weeks of intervention (T4)

The variation trends of all the assessed indicators from pre-intervention (T0), after 2 weeks of intervention (T2), to after 4 weeks of intervention (T4) are shown in Fig. 3.

No adverse events directly attributable to the intervention were observed throughout the study. Two patients in robot group and one in control group experienced an fall incidence outside of training, and there was no significant difference in the number of falls between the two groups (*P* = 1).

Results of the repeated ANOVA for all the assessed indicators are displayed in Supplementary file.

**Table 2:** Outcome measures before and after 4-week intervention in Robot group and Control group

	Robot group		Control group		Between groups	95% CI	
Variable	T0	T4	T0	T4	*P* Value	lower-bound	upper-bound
**BBS**	10.25 ± 6.47	32.5 ± 13.42*	10.92 ± 4.98	20.58 ± 12.05*	0.032*	-22.71385	-1.11949
**PASS**	16.33 ± 6.51	30.08 ± 7.74*	15.17 ± 5.34	21 ± 6.59*	0.005*	-15.16972	-2.99695
**FMA-LE**	12.33 ± 4.85	11.42 ± 4.06*	19.42 ± 6.73	16.58 ± 6.6*	0.309	-8.47786	2.81119
**MBI**	41.33 ± 12.93	62.92 ± 17.36*	41.75 ± 10.5	58.58 ± 12.8*	0.494	-17.24919	8.58252
**Tecnobody**							
**Static evaluation**							
Track length-Open eyes (mm)	229.25 ± 83.71	158.25 ± 69.18*	239.08 ± 85.26	174.5 ± 43.72*	0.5	-33.27467	65.77467
Track length-Close eyes (mm)	223.25 ± 73.38	160.25 ± 63.64*	214.61 ± 60.4	183.42 ± 71.01*	0.409	-33.92028	80.25361
Track area-Open eyes (mm^2^)	223.58 ± 282.42	47.58 ± 46.6*	237.78 ± 167.87	186.64 ± 154.93*	0.011*	38.13858	239.97808
Track area-Close eyes (mm^2^)	145.5 ± 138.59	36.42 ± 38.31*	152.19 ± 132.01	104.98 ± 116.25*	0.074	-7.56672	144.68338
**Stability limit (%)**	39.29 ± 14.97	69.89 ± 11.85*	42.7 ± 9.33	59.6 ± 9.65*	0.03*	-19.46205	-1.12128
**sEMG**							
Rectus femoris-RMS	53.85 ± 43	87.32 ± 45.74*	50.65 ± 32.81	69.1 ± 31.98*	0.272	-51.86271	15.42321
Rectus femoris-iEMG	46.42 ± 37.42	72.22 ± 38*	43.28 ± 29.05	57.69 ± 27.32*	0.295	-42.71434	13.66101
Biceps femoris-RMS	28.49 ± 21.39	44.93 ± 32.81*	25.57 ± 18.93	33.24 ± 19.27*	0.301	-34.79209	11.40209
Biceps femoris-iEMG	23.38 ± 17.53	36.24 ± 24.54*	21.03 ± 14.46	26.19 ± 13.81*	0.233	-27.1725	7.07616
Tibialis anterior-RMS	48.84 ± 78.71	55.73 ± 63.7*	44.16 ± 31.54	57.32 ± 40.11	0.942	-43.46901	46.66217
Tibialis anterior-iEMG	37.44 ± 59.38	43.94 ± 51.06	34.7 ± 25.28	41.33 ± 31.41	0.882	-38.49916	33.28233
Gastrocnemius-RMS	13.82 ± 12.73	20.93 ± 16.82	15.15 ± 15.92	22.71 ± 21.03*	0.821	-14.34029	17.89796
Gastrocnemius-iEMG	11.15 ± 9.82	16.15 ± 12.57	12.44 ± 12.51	18.05 ± 15.13*	0.741	-9.87665	13.67199

Values are presented as mean (SD). BBS, Berg Balance Function Assessment Scale; PASS, postural assessment scale for stroke; FMA-LE, Fugl–Meyer lower-extremity motor function scale; MBI, Modified Barthel Index. T0, before starting treatment; T4, after 4 weeks of treatment. *Statistically significant (*P* < 0.05).

## Discussion

In this 4-week pilot study, the combination of REX exoskeleton rehabilitation robot training with conventional rehabilitation showed improvements in balance(BBS), postural control(PASS), lower extremity motor function(FMA-LE), and static and dynamic stability in an upright position. Additionally, an increase in RMS and iEMS was observed in the rectus femoris and biceps femoris muscles, which may positively impacted the patients’ activities of daily living. Compared with the control group, the robot group can significantly improve the patient’s balance(BBS) and posture control ability(PASS).The intervention was completed by all patients without any major complications. To the best of our knowledge, this is the first study that applies the REX exoskeleton rehabilitation robot in conjunction with conventional rehabilitation training for patients with sub-acute stroke, so as to assist patients in early functional training of the trunk, upper limbs, and lower limbs in the upright position, improve their balance function and lower limb function, and enhance the efficacy and efficiency of rehabilitation.

Currently, one category of robots proposed for balance function training is the standing/sitting balance mobility training device, which consists of a standing/sitting surface, a safety range, and a monitor [29]. The training is often performed with static or dynamic stability through pelvic and trunk movements [30]. Seigo Inoue et al. demonstrated that training using the BEAR Standing Balance Function Training Robot Training System 3DBT-33 significantly improved the balance function of stroke patients [31]. Another category is the ground-based rehabilitation robot, which focuses on improving the walking ability and balance of patients by performing walking training with or without additional upper body support or assisted balance such as crutches and walking frames in the upright position [32]. Most of the existing studies suggest that the efficacy of robot assisted gait training (RAGT) is beneficial for balance function [33], especially for dynamic balance function [34]. As reported by Kim et al., the use of rehabilitation robots for trunk stability training in stroke patients was effective on improving the balance and lower limb function [35].

Notably, the REX rehabilitation robot, used in this study, focuses on upper limb and trunk functional activity training, shift of center of gravity training, and walking training in the upright position for stroke patients. Its distinctive self-balancing feature facilitates early intervention for balance and gait training in initial-stage hemiplegia. Studies have revealed the importance of rehabilitation training with repetitive functional tasks early after stroke for improving patient function [36, 37]. Our study supports the notion that rehabilitation robot training is beneficial for the balance and lower limb function in stroke patients [38]. According to our results, compared with the dose-matched control group, the robot group showed significant improvements in the balance function assessment BBS and the stroke posture scale PASS, accompanied by better static stability and stability limits in the Technology assessment index. Although there was no significant difference between the two groups in MBI, an assessment of daily living activities. We believe that the REX rehabilitation robot, effective on improving patients’ balance function and postural control, mainly aims at training patients motor function [39]. There was no training program for functional activities of daily living (e.g., washing while standing or sitting), which may explain the observation that no significant differences in MBI were observed between the two groups.This might also be attributed to the design of the experiment and the chosen assessment methods. Future evaluations might benefit from adopting the International Classification of Functioning, Disability and Health Rehabilitation Set(ICF-RS), which allows comprehensive assessment of rehabilitation improvement at the physical, individual and social levels. In this study, there was no significant difference between the two groups in lower limb motor function FMA-LE. It has been previously studied that surface rehabilitation robots do not show greater benefits for lower limb motor function FMA-LE than conventional rehabilitation [40]. There was no between-group difference in sEMG assessment of lower limb muscles (rectus femoris, biceps femoris, tibialis anterior, gastrocnemius). However, there were significant differences in RMS and iEMG of the rectus femoris and biceps femoris between the two groups after the intervention.Zhang et al. investigated the robot-assisted therapy in lower limb sEMG measurements in patients with sub-acute stroke, showing activation of muscles around the knee joints, while no noticeable changes of muscles around the ankle joint [41].

Unlike other studies, this study focused on the training effects of functional task activities combined with walking training on the balance function and postural control in stroke patients. Compared with conventional therapy, robotic gait rehabilitation can deliver highly controlled, repetitive and intensive training, reduce the physical burden for the therapist and increasing the efficiency and effectiveness of the intervention. Consistent with the outcomes of most randomized controlled trials, additional balance training is beneficial for balance function, postural control, and lower limb function in patients in the early stage of stroke [42–44]. Based on these findings, we advocate for the early use of REX rehabilitation robot in conjunction with conventional rehabilitation for the training of patients with sub-acute stroke.

Certain limitations should be noted in this study. Firstly, our study was a single center study with a small sample size of 24 patients, which might limit the generalizability of the results. Secondly, long-term follow-up results were unavailable, and only data of baseline, 2 weeks, and 4 weeks post-intervention were assessed. Thirdly, balance function is dependent on somatosensory input, central integration, and motor control, while mechanistic studies for balance improvement were lacking in this work. Lastly, the absence of a double-blind design raises the possibility of psychological biases influencing the outcomes in the robot group compared with the control group. To address these issues, future research efforts will focus on designing large-scale randomized controlled trials to further explore the impact of rehabilitation robots on balance and lower limb function, incorporating a more comprehensive methodology.

## Conclusion

REX rehabilitation robot training combined with conventional rehabilitation training promotes the balance function and postural control in patients with stroke, which is superior to training associated with the use of an upright bed. Moreover, the rehabilitation robot offers patients a safer, more effective, and engaging approach to rehabilitation. Nevertheless, further large-sample studies are warranted to investigate the effects of rehabilitation robot training on balance function, postural control, lower limb motor function, and daily living activities of patients with stroke.

### Data availability

The raw data supporting the conclusions of this article will be made available by the authors, without undue reservation.

### Ethics Statement

This study was approved by the Ethics Committee of Wuxi Mental Health Center (No. WXMHCIRB2021LLky143) and registered in the Chinese Clinical Trials Registry with the unique identifier of ChiCTR2300068398. The patients/participants provided the written informed consent to participate in this study.

### Electronic supplementary material

Below is the link to the electronic supplementary material.

Supplementary Material 1

## Data Availability

No datasets were generated or analysed during the current study.
