# Supplementary material for: Exoskeleton rehabilitation robot training for balance and lower limb function in sub-acute stroke patients: a pilot, randomized controlled trial
Source: J Neuroeng Rehabil. 2024 Jun 8;21:98. doi: 10.1186/s12984-024-01391-0 (PMC11162020; doi:10.1186/s12984-024-01391-0)
Supplement: Supplementary file 1 — Supplementary Material 1 [file 12984_2024_1391_MOESM1_ESM.docx]

## Supplementary Materials

The graphs present the statistical results of repeated ANOVA for BBS, PASS, FMA-LE, MBI, Tecnobody Balance Test, and sEMG for both groups pre-intervention (T0), and 2 weeks (T2) and 4 weeks (T4) post-intervention, respectively.

|  | T0 | T2 | T4 | Group effect | | | Time effect | | | Group*Time effect | | |
| --- | --- | --- | --- | --- | --- | --- | --- | --- | --- | --- | --- | --- |
|  | M±SD | M ± SD | M ± SD | F | P | η2 | F | P | η2 | F | P | η2 |
| **Berg** |  |  |  |  |  |  |  |  |  |  |  |  |
| Robot group | 10.25 ± 6.47 | 21.83 ± 12.04 | 32.5 ± 13.42 | 1.908 | 0.181 | 0.08 | 53.4 | < 0.001* | 0.708 | 8.34 | 0.005* | 0.275 |
| Control group | 10.92 ± 4.98 | 17.33 ± 9.91 | 20.58 ± 12.05 |  |  |  |  |  |  |  |  |  |
| **PASS** |  |  |  |  |  |  |  |  |  |  |  |  |
| Robot group | 16.33 ± 6.51 | 25.25 ± 6.15 | 30.08 ± 7.74 | 7.028 | 0.015* | 0.242 | 15.68 | < 0.001* | 0.579 | 2.4 | 0.027* | 0.19 |
| Control group | 15.17 ± 5.34 | 18.42 ± 5.62 | 21 ± 6.59 |  |  |  |  |  |  |  |  |  |
| **FMA-LE** |  |  |  |  |  |  |  |  |  |  |  |  |
| Robot group | 12.33 ± 4.85 | 16.58 ± 5.87 | 19.42 ± 6.73 | 1.287 | 0.269 | 0.055 | 28.73 | < 0.001* | 0.084 | 2.006 | 0.162 | 0.655 |
| Control group | 11.42 ± 4.06 | 13.08 ± 4.64 | 16.58 ± 6.6 |  |  |  |  |  |  |  |  |  |
| **MBI** |  |  |  |  |  |  |  |  |  |  |  |  |
| Robot group | 41.33 ± 12.93 | 53.00 ±14.47 | 62.92 ± 17.36 | 0.185 | 0.671 | 0.008 | 81.137 | < 0.001* | 0.787 | 1.296 | 0.274 | 0.056 |
| Control group | 41.75 ± 10.5 | 50.17 ± 11.86 | 58.58 ± 12.8 |  |  |  |  |  |  |  |  |  |
| **Static evaluation** | |  |  |  |  |  |  |  |  |  |  |  |
| Track length-Open eyes (mm) | |  |  |  |  |  |  |  |  |  |  |  |
| Robot group | 229.25 ± 83.71 | 161.58 ± 49.11 | 158.25 ± 69.18 | 0.77 | 0.39 | 0.034 | 25.75 | < 0.001* | 0.71 | 2.224 | 0.133 | 0.175 |
| Control group | 239.08 ± 85.26 | 204.5 ± 70.73 | 174.5 ± 43.72 |  |  |  |  |  |  |  |  |  |
| Track length-Close eyes (mm) | |  |  |  |  |  |  |  |  |  |  |  |
| Robot group | 223.25 ± 73.38 | 160.17 ± 36.51 | 160.25 ± 63.64 | 0.224 | 0.528 | 0.018 | 15.109 | < 0.001* | 0.59 | 2.478 | 0.108 | 0.191 |
| Control group | 214.61 ± 60.4 | 192.25 ± 67.85 | 183.42 ± 71.01 |  |  |  |  |  |  |  |  |  |
| Track area-Open eyes (mm^2^) | |  |  |  |  |  |  |  |  |  |  |  |
| Robot group | 223.58 ± 282.42 | 77.08 ± 73.05 | 47.58 ± 46.6 | 2.804 | 0.108 | 0.113 | 7.689 | 0.011* | 0.259 | 2.959 | 0.098 | 0.119 |
| Control group | 237.78 ± 167.87 | 218.78 ± 166.44 | 186.64 ± 154.93 |  |  |  |  |  |  |  |  |  |
| Track area-Close eyes (mm^2^) | |  |  |  |  |  |  |  |  |  |  |  |
| Robot group | 145.5 ± 138.59 | 42.17 ± 48.06 | 36.42 ± 38.31 | 1.846 | 0.188 | 0.077 | 16.351 | < 0.001* | 0.426 | 4.542 | 0.04* | 0.171 |
| Control group | 152.19 ± 132.01 | 132.5 ± 124.29 | 104.98 ± 116.25 |  |  |  |  |  |  |  |  |  |
| **Stability limit (%)** | |  |  |  |  |  |  |  |  |  |  |  |
| Robot group | 39.29 ± 14.97 | 55.64 ± 10.31 | 69.89 ± 11.85 | 1.183 | 0.288 | 0.051 | 59.219 | < 0.001* | 0.849 | 4.755 | 0.02 | 0.312 |
| Control group | 42.7 ± 9.33 | 49.65 ± 9.35 | 59.6 ± 9.65 |  |  |  |  |  |  |  |  |  |
| **Rectus femoris** | |  |  |  |  |  |  |  |  |  |  |  |
| RMS |  |  |  |  |  |  |  |  |  |  |  |  |
| Robot group | 53.85 ± 43 | 79.71 ± 53.97 | 87.32 ± 45.74 | 0.43 | 0.519 | 0.019 | 17.795 | < 0.001* | 0.629 | 1.033 | 0.373 | 0.09 |
| Control group | 50.65 ± 32.81 | 69.38 ± 41.76 | 69.1 ± 31.98 |  |  |  |  |  |  |  |  |  |
| iEMG |  |  |  |  |  |  |  |  |  |  |  |  |
| Robot group | 46.42 ± 37.42 | 65.92 ± 44.61 | 72.22 ± 38 | 0.334 | 0.569 | 0.015 | 13.954 | < 0.001* | 0.571 | 0.732 | 0.493 | 0.065 |
| Control group | 43.28 ± 29.05 | 59.95 ± 34.61 | 57.69 ± 27.32 |  |  |  |  |  |  |  |  |  |
| **Biceps femoris** | |  |  |  |  |  |  |  |  |  |  |  |
| RMS |  |  |  |  |  |  |  |  |  |  |  |  |
| Robot group | 28.49 ± 21.39 | 37.07 ± 23.82 | 44.93 ± 32.81 | 0.83 | 0.372 | 0.036 | 7.216 | 0.004* | 0.407 | 1.157 | 0.334 | 0.099 |
| Control group | 25.57 ± 18.93 | 28.06 ± 18.54 | 33.24 ± 19.27 |  |  |  |  |  |  |  |  |  |
| iEMG |  |  |  |  |  |  |  |  |  |  |  |  |
| Robot group | 23.38 ± 17.53 | 29.16 ± 19 | 36.24 ± 24.54 | 0.855 | 0.365 | 0.037 | 6.541 | 0.006* | 0.384 | 1.203 | 0.32 | 0.103 |
| Control group | 21.03 ± 14.46 | 23.2 ± 13.57 | 26.19 ± 13.81 |  |  |  |  |  |  |  |  |  |
| **Tibialis anterior** | |  |  |  |  |  |  |  |  |  |  |  |
| RMS |  |  |  |  |  |  |  |  |  |  |  |  |
| Robot group | 48.84 ± 78.71 | 46.7 ± 61.25 | 55.73 ± 63.7 | 0.001 | 0.973 | 0 | 1.918 | 0.177 | 0.08 | 0.458 | 0.538 | 0.02 |
| Control group | 44.16 ± 31.54 | 52.01 ± 33.63 | 57.32 ± 40.11 |  |  |  |  |  |  |  |  |  |
| iEMG |  |  |  |  |  |  |  |  |  |  |  |  |
| Robot group | 37.44 ± 59.38 | 37.36 ± 49.47 | 43.94 ± 51.06 | 0.012 | 0.914 | 0.001 | 1.374 | 0.258 | 0.059 | 0.06 | 0.844 | 0.003 |
| Control group | 34.7 ± 25.28 | 37.2 ± 27.43 | 41.33 ± 31.41 |  |  |  |  |  |  |  |  |  |
| **Gastrocnemius** | |  |  |  |  |  |  |  |  |  |  |  |
| RMS |  |  |  |  |  |  |  |  |  |  |  |  |
| Robot group | 13.82 ± 12.73 | 18.99 ± 17.09 | 20.93 ± 16.82 | 0 | 1 | 0 | 4.793 | 0.019* | 0.31 | 0.985 | 0.39 | 0.086 |
| Control group | 15.15 ± 15.92 | 15.88 ± 10.08 | 22.71 ± 21.03 |  |  |  |  |  |  |  |  |  |
| iEMG |  |  |  |  |  |  |  |  |  |  |  |  |
| Robot group | 11.15 ± 9.82 | 14.68 ± 12.27 | 16.15 ± 12.57 | 0.012 | 0.912 | 0.001 | 4.31 | 0.027* | 0.291 | 0.924 | 0.412 | 0.081 |
| Control group | 12.44 ± 12.51 | 12.98 ± 6.78 | 18.05 ± 15.13 |  |  |  |  |  |  |  |  |  |

*Statistically significant (P < 0.05).

The data were tested by S-W (Shapiro-Wilk) test and conformed to normal distribution.

According to repeated ANOVA results for BBS scores, there was an interaction effect between group and time point of assessment (p < 0.05). In addition, results of simple effects test for the group showed a non-significant group effect at T0 (F = 0.08, p = 0.78, skewed η2 = 0.004) and T2 (F = 1.00, p = 0.328, skewed η2 = 0.043), and a significant group effect at T4 (F = 5.24, p < 0.05, skewed η2=0.19). Besides, based on results of simple effects test for assessment time points, the effects of robot group (F=33.15, p<0.05, skewed η2=0.76) and control group (F=5.40, p<0.05, skewed η2=0.34) were significant across the different assessment time points. In addition, significant differences were detected at T0 (p < 0.05), T2 (p < 0.05) and T4 (p < 0.05) in robot group; there were significant differences at T2 (p < 0.05) and T4 (p < 0.05) in control group, while no significant difference was found between T2 and T4 (p = 0.076).

Results of repeated ANOVA for PASS scores demonstrated the presence of an interaction effect between group and time point of assessment (p < 0.05). Results of the simple effects for the group indicated a non-significant simple effect for group at T0 (F = 0.23, p = 0.64, skewed η2 = 0.01); T2 (F = 8.08, p < 0.05, skewed η2 = 0.269) and T4 (F = 9.58, p < 0.05, skewed η2 = 0.269). In addition, results of simple effects test for measurement time points indicated that the robot group (F=15.67, <0.05, skewed η2=0.60) exhibited a significant simple effect for different assessment time points; while the control group (F=3.05, p=0.069, skewed η2=0.23) did not exhibit a significant simple effect for different assessment time points. Differences at T0, T2 and T4 reached the significance level in the robot group (p < 0.05). In control group, there was no significant difference at T2 (p = 0.187) or T4 (p = 0.071) compared with T0, and the difference between T2 and T4 was not significant (p = 0.080). Both FMA-LE and MBI showed statistically significant differences across different assessment time points (p < 0.05), but there was no interaction between group and assessment time point (p > 0.05).

Results of balance function tests performed by the Tecnobody Balance Tester, including the items assessed for static stability (the area of trajectory with eyes open and closed, and the length of movement trajectory with eyes opened and closed), as well as the item assessed for dynamic balance function (limit of stabilization). As revealed by our repeated ANOVA results, there was an interaction effect between group and assessment time point (p<0.05). Besides, group effect test results revealed no significant group effects at T0 (F=0.015, p=0.905, skewed η2=0.001) and T4 (F=3.765, p=0.065, skewed η2=0.146), but significant group effect was detected at T2 (F=5.514, p<0.05, skewed η2=0.200). The test results for assessment time point effects indicated significant effects of assessment time points in robot group (F=9.164, p<0.05, skewed η2=0.466) and control group (F=8.120, p<0.05, skewed η2=0.436). In robot group, the effects at T2 (p< 0.05) and T4 (p < 0.05) were significantly different from that at T0, whereas there was no significant difference in PASS values between T2 and T4 (p = 1). For control group, differences at T2 (p = 0.187) and T4 (p = 0.071) were not significant compared with T0, but there was a significant difference between T2 and T4 (p < 0.05). The assessments of movement trajectory and movement length with eyes open, movement length with eyes closed, and stable limits were all significant for the effect of assessment time (p < 0.05), but there was no interaction between group and assessment time (p > 0.05).

Results of repeated ANOVA for sEMG assessment demonstrated a significant effect of assessment time for RMS and iEMG of the rectus femoris, biceps femoris, and gastrocnemius muscles (p < 0.05), but there was no statistically significant interaction between group and assessment time (p > 0.05). The effects of group, time and group-time interaction were not statistically significant for RMS and iEMG of tibialis anterior muscle (p > 0.05).
